# Hospitalizations for cancer in international migrants *versus* local population in Chile

**DOI:** 10.11606/S1518-8787.2018052000222

**Published:** 2018-03-14

**Authors:** Marcela Oyarte, Iris Delgado, Víctor Pedrero, Lorenzo Agar, Báltica Cabieses

**Affiliations:** I Universidad del Desarrollo. Facultad de Medicina Clínica Alemana. Santiago, Chile; II Universidad de Chile. Facultad de Medicina. Santiago, Chile; III University of York. Department of Health Sciences. York, England

**Keywords:** Emigrants and Immigrants, Neoplasms, epidemiology, Hospitalization, Health Services Accessibility, Socioeconomic Factors, Health Inequalities, Latin America, Emigrantes e Inmigrantes, Neoplasias, epidemiología, Hospitalización, Accesibilidad a los Servicios de Salud, Factores Socioeconómicos, Desigualdades en la Salud, América Latina

## Abstract

**OBJECTIVE:**

To compare cancer hospital morbidity among the local population and the immigrant population in Chile.

**METHODS:**

This is a prevalence study based on the analysis of hospital discharges of all the health centers of Chile. Cancer hospital discharges were characterized in 2012 according to the migratory status. The crude and specific rates of hospital morbidity for this cause were estimated for the analysis of their association with migratory status using zero-inflated negative binomial regression, adjusted for sociodemographic variables.

**RESULTS:**

The neoplasms were the third cause of hospital discharges for immigrants and the seventh one for Chileans. The adjusted rate of cancer hospital discharges was higher for Chileans than immigrants, and the latter had fewer days of hospitalization and greater proportion of surgical interventions. In the group of immigrants, cancer hospital discharges mainly corresponded to patients belonging to the private system (46%), and in the group of Chileans they mainly corresponded to patients in the public system (71.1%). We observed a large difference in the proportion of cancer hospital discharges for patients with no health insurance between the two populations (22.6%: immigrants, 1.0%: Chileans). In both populations, the three most frequent types of cancer were: (i) lymphoid tissue, hematopoietic organs, and related tissues, (ii) digestive organs, and (iii) breast cancer.

**CONCLUSIONS:**

Models of differentiated care should be considered for immigrants, with the creation of specific programs of information, coverage, and protection against cancer. More information on this problem must be generated at the local and international level.

## RESUMEN

**OBJETIVO:**

Comparar la morbilidad hospitalaria por cáncer entre población local e inmigrante en Chile.

**MÉTODOS:**

Estudio de prevalencia basado en el análisis de egresos hospitalarios de todos los centros de Chile. Se caracterizaron los egresos hospitalarios por cáncer en 2012 según condición migratoria. Se estimaran las tasas brutas y específicas de morbilidad hospitalaria por esta causa, para finalmente analizar su asociación con el estatus migratorio mediante una regresión binomial negativa inflada por ceros ajustada por variables sociodemográficas.

**RESULTADOS:**

Las neoplasias fueron la tercera causa de egresos hospitalarios en inmigrantes y la séptima en chilenos. La tasa ajustada de egresos hospitalarios por cáncer fue mayor en chilenos que en inmigrantes, y estos presentaron menor cantidad de días de hospitalización y mayor proporción de intervenciones quirúrgicas. En el grupo de inmigrantes, los egresos hospitalarios por cáncer corresponderán principalmente a pacientes pertenecientes al sistema privado (46%) y en chilenos a pacientes en el sistema público (71,1%). Se observó una amplia diferencia en la proporción de egresos hospitalarios por cáncer correspondientes a pacientes sin previsión de salud entre ambas poblaciones (22,6%: inmigrantes, 1,0%: chilenos). En ambas poblaciones, los tres cánceres que se presentaron con mayor frecuencia fueron: (i) tejidos linfáticos, órganos hematopoyéticos y tejidos afines, (ii) órganos digestivos y (iii) cáncer de mama.

**CONCLUSIONES:**

Se deben considerar modelos de atención diferenciada en inmigrantes, creando programas específicos de información, cobertura y protección frente al cáncer. Es necesario generar más información sobre esta problemática a nivel local e internacional.

## INTRODUCTION

Globally, close to 200 million persons live in a place different from where they were born^1^. In Latin America, it was estimated that approximately 4.0% of the population lived in a country other than that of origin in 2011^2^. The immigrant population usually presents a population structure, in sex, age, and other factors, that is different from the local population^3^.

For all the changes that the migratory process implies both for the individuals, by modifying their conditions and lifestyles, and for the population, given the patterns of infectious and chronic diseases that individuals bring with them, international migration has the potential to generate high impact on public health^4^. In Chile^3^ and in the world^5^ international migration has been recognized as a valuable indicator of vulnerability and social inequality in health between and within countries^3–5^.

International migration itself does not necessarily pose a threat to mental and general health. Its association with factors such as cultural barriers, difficulty of access and use of health services, conditions of the country of origin, residence time, and social, economic, labor, and educational factors^6^ ^,^ ^7^, among others, determines the individual and family well-being of the immigrant. It is in relation to these factors that it becomes important to link the evidence on international migration, health, and social vulnerability. Similarly, it is important to analyze the health and use of services by this population for all levels of health care. Immigrants can report different expectations, problems, and needs related to health care^8^. The international literature shows that: (i) there is insufficient use of health services by immigrants compared to the local population in some countries, (ii) in general, most immigrants use public health services^9^, and (iii) secondary care services are used by immigrants in some countries, in some cases without even having used the primary level, with the problem that they have a limited role in the provision of services^7^.

It is thus that diseases, for which secondary prevention and adequate treatment can be determinant to reduce their incidence and mortality, become a focus of special care for immigrants. Cancer fulfills these characteristics^10^ and is a global public health problem because of its high therapeutic cost and high burden as a disease^11^. In the Americas, 2.8 million new cases were registered in 2012, of which approximately 47% occurred in Latin America and the Caribbean^12^.

Cancer is a health priority in Chile, presenting high prevalence, mortality, and heterogeneity throughout the country^13^. Although it has been studied extensively for the local population^14^, the information on cancer in immigrants in the country is scarce^10^. The distribution of cancer prevalence, risk, and mortality differs between local and immigrant population and according to the characteristics of these populations in each country. In Italy, the risk of colorectal^15^ or cervical cancer^16^ is higher in the immigrant population compared to the local population. On the other hand, no differences have been observed in Denmark between the local and foreign population in cancer mortality^17^. In Spain, local women had higher rates of detection of cervical cancer than immigrants^10^, and the latter were less likely to undergo gynecological cancer screening^18^.

In Italy, despite the total hospitalization rates being higher for the local population than the immigrant population, it was the latter one that presented a higher rate of cervical cancer and a lower rate of mastectomies^19^. Often, cancer is not the leading cause of death in international migrants. However, in countries such as Sweden, cancer is the third cause of death in this population^20^, highlighting the importance of studying this phenomenon globally and in countries with a high health burden for this disease.

The objective of this study was to compare cancer hospital morbidity among the local population and immigrant population in Chile. We aimed to contribute with current national and international knowledge on the health of the immigrant population, especially focusing on hospitalizations for cancer.

## METHODS

This is a prevalence study based on the analysis of secondary data from hospital discharges of all public and private centers in Chile, compiled by the Department of Health Statistics and Information (DEIS) of the Ministry of Health of Chile, in 2012. This year was the first time when information on the nationality of the subject treated at the hospitals was registered. For the purposes of this study, we considered as synonyms the concepts of foreign population (nationality other than Chilean) and immigrants or international migrants. Cancer hospital discharges (CHD) were characterized in 2012 according to migratory status. We studied the crude and specific rates of hospital morbidity for this cause to analyze the association between them and migratory status (Chilean or immigrant). The analysis of hospital discharges provides information on both morbidity and health care services, according to the pathology explored^19^. The information obtained from them represents a strong indicator of morbidity of a country, even if we exclude the discharges for obstetric causes. Therefore, their analysis allows knowing and adjusting the health services to the needs of different subgroups of the population (in this case, the immigrant population) at the primary and secondary level^21^.

The database of hospital discharges in Chile in 2012 contains: (i) demographic characteristics (sex, age, nationality, place of residence), (ii) health insurance (public, private, none, others), and (iii) characteristics of the hospitalization, such as diagnosis (coded according to ICD-10), condition of the discharges (discharge or death), surgical intervention (yes, no), days of hospitalization, date, and information on the center where the hospital discharge occurred. We estimated the absolute numbers and those relative to migratory status from the characteristics mentioned above in hospital discharges, whose main diagnosis corresponded to the codes C00-D09 (*in situ* and malignant neoplasms) of ICD-10.

The specific rates of cancer hospital morbidity (CHM) were amplified by 100,000 and were calculated as the ratio between the number of hospital discharges per *in situ* and malignant neoplasms in the subgroups of: migratory status, age, sex, and type of health insurance, in 2012 (numerator), and total population in the same subgroup for the same period (denominator). The number of hospital discharges was obtained using its homonymous database and the denominators of the population database, that is, the total population by subgroup, from the average of the estimates of population obtained using the studies of national socioeconomic characterization (CASEN) in the 2011 and 2013 versions. Both versions of the research use a complex sampling, which was considered for the estimates and which are representative of the residents in private households in the fifteen regions of the country excluding areas of difficult access and institutionalized persons^22^.

The association between cancer hospital morbidity and migratory status was analyzed using an explanatory model of zero-inflated negative binomial regression (ZINB). In this model, the count values are generated from a negative binomial model and the excess zeros come from a binary model. It is suitable for situations that have excess zeros and dispersion, in addition to the assumption that the event is not impossible in any of the populations under study or in their subgroups^23^, as is the case with CHD.

The main dependent variable for this analysis was the specific rate of cancer hospital morbidity and the main independent variable was the migratory status. Based on the literature and availability, we considered the confounding variables: sex, age, health insurance, and the interaction between the variables of migratory status and health insurance, resulting in a model as: λ

log (λ) ~ log (p) + migratory status + sex + age + age^2^ + insurance + migratory status insurance

logit (π) = log (1/1-π) ~ migratory status + age + sex + health insurance

Where corresponds to the number of hospital discharges in the subgroup, corresponds to the logarithm of the total population of the country in the subgroup (offset), and corresponds to the probability of observing excess zero. The fit tests of the model were corroborated.

## RESULTS

Approximately 74,282 CHD happened in Chile in 2012, which is equivalent to 4.5% of the total HD of the country in the period. Of these, 0.7% corresponded to the immigrant population. Neoplasms (C00-D48 of ICD-10) corresponded to the third most common cause of hospital discharges in immigrants; on the other hand, neoplasms corresponded to the seventh most common cause in the local population, according to the large chapters of causes of the ICD-10.

The three most common causes of CHD in both the local and immigrant population were malignant neoplasms of: (i) digestive organs (22.9% total: 18.5% immigrants, 22.9% Chileans, codes C15-C26 of ICD-10), (ii) lymphoid tissue, hematopoietic organs, and related tissues (17.4% total: 17.7% immigrants, 17.4% Chileans, codes C81-C96 of ICD-10), and (iii) breast (10.3% total: 16.7% immigrants, 10.3% Chileans, code C50 of ICD-10) (Table 1).

**Table 1:** Hospital profile of patients who were discharged because of cancer (C00-D09 of ICD-10), according to migratory status. Chile, 2012.

Hospital characteristics	Chilean (n = 74,382)		Immigrant (n = 486)		Total (n = 74,868)	
		
	n	%	n	%	n	%
Type of cancer						
Digestive organs	17,042	22.9	90	18.5	17,132	22.9
Lymphoid tissue, hematopoietic organs, and related tissues	12,904	17.4	86	17.7	12,990	17.4
Breast	7,647	10.3	81	16.7	7,728	10.3
Female genital organs	6,289	8.5	36	7.4	6,325	8.4
Ill-defined, other secondary, and unspecified sites	3,655	4.9	35	7.2	3,690	4.9
Respiratory and intrathoracic organs	3,789	5.1	34	7.0	3,823	5.1
*In situ*	3,029	4.1	31	6.4	3,060	4.1
Thyroid gland and other endocrine glands	3,789	5.1	22	4.5	3,811	5.1
Male genital organs	5,694	7.7	17	3.5	5,711	7.6
Lips, oral cavity, and pharynx	995	1.3	16	3.3	1,011	1.4
Melanoma and other malignant neoplasms of skin	2,052	2.8	14	2.9	2,066	2.8
Urinary tract	3,068	4.1	12	2.5	3,080	4.1
Eye, brain, and other parts of central nervous system	2,049	2.8	8	1.6	2,057	2.7
Mesothelial and soft tissue	1,129	1.5	3	0.6	1,132	1.5
Bone and articular cartilage	1,005	1.4	1	0.2	1,006	1.3
Malignant neoplasms of independent (primary) multiple sites	146	0.2	0	0	146	0.2
Discharge condition						
Alive	70,446	94.8	474	97.5	70,920	94.9
Deceased	3,836	5.2	12	2.5	3,848	5.1
Surgical intervention						
Yes	30,022	40.4	210	43.2	30,232	40.4
No	44,260	59.6	276	56.8	44,536	59.6
Days of hospitalization						
1 day	19,202	25.8	183	37.7	19,385	25.9
2 days	10,441	14.0	89	18.3	10,530	14.1
3 days	7,473	10.0	55	11.3	7,528	10.1
4 or more days	37,266	50.1	159	32.7	37,425	50.0
Average (standard deviation)	7.5 (12.3)		5.9 (15.3)		7.5 (12.4)	
Minimum/Maximum	1/349		1/235		1/349	

The local population presented a cancer hospital mortality rate that was almost double that of immigrants (5.2% Chileans, 2.5% immigrants) (Table 1). The latter had a lower average age of death (60 years for immigrants *versus* 65 years for Chileans), increasing their years of potential life lost. No death was registered within the HD in older adults aged more than 75 years for immigrants. In contrast, we found 30% of the total CHD for this age range reported as deceased for Chileans.

Chileans presented 50.1% of the CHD in patients with four or more days of stay, while this percentage was 32.7% for immigrants. Conversely, for surgical interventions (SI), the proportion is 0.3 percentage points higher in the immigrant population (Table 1). This situation was maintained for the different types of cancer, going from two to seventy-one percentage points of difference between Chileans and immigrants. The exceptions were the neoplasms of breast, female organs, *in situ*, and ill-defined sites, in which Chileans had a higher percentage of SI than immigrants. Breast cancer was the only type that presented a statistically significant difference in the local population (72.3% Chileans, 46.9% immigrants). We also observed differences in the proportion of surgical interventions, and only the private system (Institutions of Health Insurance [ISAPRE]) registered a higher percentage of SI in Chileans than in immigrants (46.8% Chileans, 46.9% immigrants, statistically significant difference) (Figure).

**Figure:**
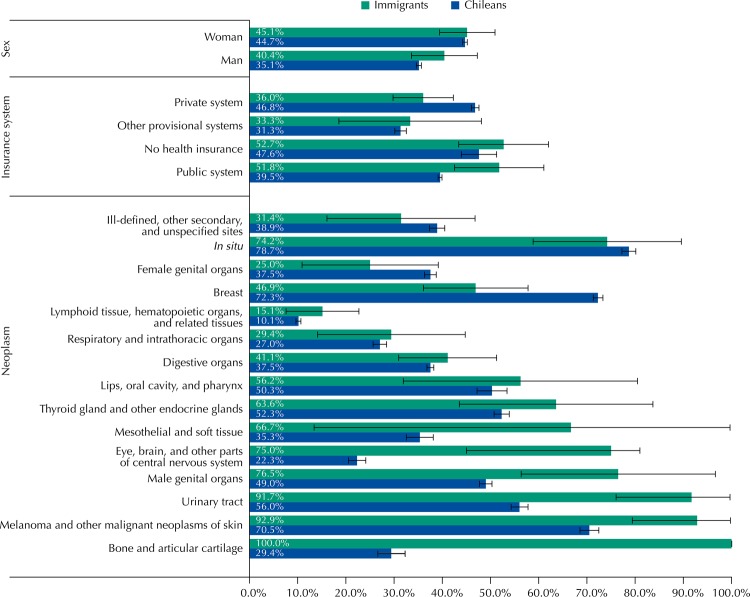
Percentage of surgical interventions in cancer patients (C00-D09 of the ICD-10) with the respective confidence intervals (95% confidence level), for Chileans and immigrants, according to the type of neoplasm, sex, and health insurance. Chile, 2012.

We observed the most evident differences between Chileans and immigrants for health insurance. For Chileans, the highest proportion of CHD, close to 70.0%, occurred in patients with National Health Fund (FONASA), even after stratifying by sex and age. In contrast, for immigrant population, the highest proportion occurred in patients with ISAPRE (46.3%), even after stratifying by sex. We highlight the high percentage of CHD for immigrants without health insurance, 22.6% (equivalent to 110 CHD), which is above the 1.0% of CHD (equivalent to 742 CHD) for Chileans in the same situation. For both populations, women had more CHD than men. However, this situation is not transversal for all age groups; for example, in children under 14 years of age, the proportion of CHD for men was higher (Table 2).

**Table 2:** Sociodemographic profile (sex, age, health insurance) of patients who were discharged because of cancer (C00-D09 of ICD-10), according to migratory status. Chile, 2012

Migratory status/ sex/ health insurance^d^			Age (years)^a^							

			0 to 14 (n = 6,370)^b^	15 to 29 (n = 4,702)	30 to 44 (n = 9,512)	45 to 59 (n = 19,215)	60 to 74 (n = 23,565)	75 to 89 (n = 10,377)	90 or more (n = 541)	Total (n = 74,282)
Chilean	Man	Public	81.1%	72.8%	60.9%	66.2%	69.3%	73.5%	67.9%	70.1%
		Private	11.3%	19.6%	30.6%	25.7%	20.5%	13.2%	10.7%	20.2%
		No insurance	0.4%	0.8%	1.3%	1.1%	1.2%	1.0%	1.3%	1.0%
		Other	7.2%	6.9%	7.2%	7.1%	9.0%	12.3%	20.1%	8.7%
		Total	100%	100%	100%	100%	100%	100%	100%	100%
		% of men^c^	56.3%c	52.0%	31.4%	36.3%	49.9%	49.5%	41.4%	44.6%
	Woman	Public	75.1%	71.0%	67.4%	68.8%	73.8%	78.7%	78.2%	71.9%
		Private	13.5%	20.0%	26.7%	24.4%	18.6%	10.1%	4.4%	20.2%
		No insurance	0.4%	1.5%	1.0%	0.8%	0.9%	1.1%	2.2%	0.9%
		Other	11.1%	7.5%	5.0%	6.1%	6.6%	10.1%	15.1%	7.1%
		Total	100%	100%	100%	100%	100%	100%	100%	100%
	Total	Public	78.4%	71.9%	65.3%	67.9%	71.6%	76.1%	73.9%	71.1%
		Private	12.3%	19.8%	27.9%	24.8%	19.6%	11.7%	7.0%	20.2%
		No insurance	0.4%	1.1%	1.1%	0.9%	1.1%	1.1%	1.8%	1.0%
		Other	8.9%	7.1%	5.7%	6.4%	7.8%	11.2%	17.2%	7.8%
		Total	100%	100%	100%	100%	100%	100%	100%	100%
			0 to 14	15 to 29	30 to 44	45 to 59	60 to 74	75 to 89	90 or more	Total
			(n = 18)	(n = 19)	(n = 78)	(n = 143)	(n = 154)	(n = 71)	(n = 3)	(n = 486)
Immigrant	Man	Public	58.3%	33.3%	9.1%	22.0%	10.8%	17.6%	100.0%	19.2%
		Private	16.7%	0.0%	36.4%	52.0%	37.3%	47.1%	0%	39.9%
		No insurance	25.0%	66.7%	54.5%	22.0%	36.1%	29.4%	0%	32.3%
		Other	0%	0%	0%	4.0%	15.7%	5.9%	0%	8.6%
		Total	100%	100%	100%	100%	100%	100%	100%	100%
		% of menb	66.7%	31.6%	14.1%	35.0%	53.9%	47.9%	66.7%	40.7%
	Woman	Public	0%	69.2%	40.3%	22.6%	16.9%	13.5%	0%	25.7%
		Private	33.3%	15.4%	56.7%	45.2%	50.7%	70.3%	0%	50.7%
		No insurance	66.7%	7.7%	3.0%	21.5%	19.7%	10.8%	100%	16.0%
		Other	0%	7.7%	0%	10.8%	12.7%	5.4%	0%	7.6%
		Total	100%	100%	100%	100%	100%	100%	100%	100%
	Total	Public	38.9%	57.9%	35.9%	22.4%	13.6%	15.5%	66.7%	23.0%
		Private	22.2%	10.5%	53.8%	47.6%	43.5%	59.2%	0%	46.3%
		No insurance	38.9%	26.3%	10.3%	21.7%	28.6%	19.7%	33.3%	22.6%
		Other	0%	5.3%	0%	8.4%	14.3%	5.6%	0%	8.0%
		Total	100%	100%	100%	100%	100%	100%	100%	100%

^a^ Of the hospital discharges for Chileans, 8.6% were patients aged between 1 and 14 years, 6.3% between 15 and 29, 12.8% between 30 and 44, 25.9% between 45 and 59, 31.7% between 60 and 74, 14% between 75 and 89, and 0.7% were aged 90 years or more. Of the hospital discharges for immigrants, 3.7% were patients aged between 1 and 14 years, 3.9% between 15 and 29, 16% between 30 and 44, 29.4% between 45 and 59, 31.7% between 60 and 74, 14.6% between 75 and 89, and 0.6% were aged 90 years or more.

^b^ “n” corresponds to the total of Chileans or immigrants, as appropriate, in the age group.

^c^ Of the hospital discharges corresponding to Chilean patients aged between 0 and 14 years, 56.3% were men and, consequently, 43.7% were women. An analog reading can be carried out for all percentages present in this row of % of men.

^d^ The National Health Fund (FONASA) corresponds to the Public System, and the Institutions of Health Insurance (ISAPRE) corresponds to the private health system.

The information provided up to this point shows the characteristics of hospitalization and the (absolute and relative) frequency of CHD in both populations and their subgroups. However, in order to understand the dynamics of the CHD, we need to analyze them also in terms of rates (crude and specific), especially if we wish to compare populations with different structures, as is the case of Chileans and immigrants.

The 486 CHD for immigrants suppose a rate of 162.4 discharges per 100,000 persons; the 74,282 CHD for Chileans suppose a rate of 446.6 discharges per 100,000 persons. Standardized by sex and age, immigrants had a rate of 249.3 CHD per 100,000 persons and the local population had a rate of 446 CHD per 100,000 persons. In both cases, Chileans presented a higher rate of CHD, but when we standardize it, the gap decreases between both populations.

The specific rates by sex and age groups were higher for Chileans than for immigrants. This situation was repeated for the different types of health insurance, with the exception of the subgroup that had no insurance (174.6 × 100,000 persons for Chileans, 335.2 × 100,000 persons for immigrants). Among men without health insurance, the rates of CHD were higher for immigrants than for the local population for all age subgroups; on the other hand, in the case of women without health insurance, this situation occurred only in those aged between zero and 14, 45 and 74, and older adults aged over 90 years. In immigrants younger than 15 years of age, although the amount of CHD corresponding to patients from FONASA and without insurance was the same (n = 8), the rate was higher in those without insurance with 98.9 CHD per 100,000 persons (Table 3).

**Table 3:** Specific rates of cancer hospital morbidity according to sex, age group, migratory status, and health insurance, amplified by 100,000. Chile, 2012.

Variable		Chilean 446.6*					Immigrant 162.4*				
	
		Public	Private	Other	None	Total	Public	Private	Other	None	Total
										
Sexo	Age (years)	397.0	669.0	1279.0	174.6	446.6	56.0	411.8	629.3	335.2	162.4
Man	0 to 14	198.5	179.1	568.2	66.0	201.8	58.4	78.6	0.0	66.0	61.8
	15 to 29	116.5	142.5	267.1	22.9	118.8	7.2	0.0	0.0	68.9	13.5
	30 to 44	176.1	331.2	538.6	55.5	205.7	3.9	48.9	0.0	137.3	27.0
	45 to 59	417.3	808.4	1072.1	127.9	478.6	101.5	436.5	585.7	796.2	263.1
	60 to 74	1162.4	3058.9	3598.1	870.1	1407.8	200.6	2080.5	4788.2	3236.2	1138.1
	75 to 89	1448.6	4790.9	4508.7	2312.4	1746.8	319.5	2608.0	1126.8	8368.2	1186.9
	90 or more	885.9	1390.9	3440.4	707.5	1072.4	489.6	0	-	-	455.6
	Total	379.7	580.2	1200.6	136.6	419.4	45.7	300.4	473.1	373.3	147.6
Woman	0 to 14	146.0	165.4	794.0	45.4	159.8	0.0	61.9	0.0	157.8	28.2
	15 to 29	95.9	158.4	385.0	59.8	108.3	23.9	26.6	159.5	14.8	24.6
	30 to 44	332.4	702.5	1056.4	203.8	396.3	70.7	354.7	0.0	60.4	125.0
	45 to 59	599.2	1320.8	1712.0	278.1	710.2	127.6	851.6	2285.7	958.1	386.1
	60 to 74	944.7	2753.7	2329.4	1146.4	1121.2	185.7	2138.4	4109.6	2737.0	799.0
	75 to 89	1045.2	2682.8	2915.2	1290.8	1184.4	194.9	9647.5	784.3	1027.0	1059.6
	90 or more	612.8	1400.0	1196.1	958.9	676.4	0	-	0	1550.4	120.9
	Total	411.8	763.4	1367.2	235.0	471.2	63.3	515.2	844.9	293.5	174.4
Total	0 to 14	172.6	172.2	672.0	55.1	181.0	26.2	69.3	0.0	98.9	44.2
	15 to 29	105.8	149.8	315.4	37.9	113.5	16.8	13.3	39.7	39.8	19.5
	30 to 44	263.8	506.9	763.4	99.5	306.9	43.9	222.2	0.0	104.1	82.7
	45 to 59	519.0	1067.2	1383.6	183.7	604.1	117.2	624.5	1540.4	893.6	331.8
	60 to 74	1038.8	2905.3	2923.3	970.6	1248.1	191.8	2111.2	4485.2	3058.7	951.8
	75 to 89	1205.7	3565.1	3609.2	1643.9	1409.2	247.6	4756.5	924.9	2750.5	1117.0
	90 or more	694.1	1394.2	1747.8	866.6	798.5	173.0	0	0	1550.4	237.0
	Total	397.0	669.0	1279.0	174.6	446.6	56.0	411.8	629.3	335.2	162.4

* The standardized rate of cancer hospital morbidity for Chileans is 446 (95%CI 404.7–487.3) and the standardized rate of hospital cancer morbidity for immigrants is 249.3 (95%CI 218.4–280.2). The rates were standardized using the total population of Chile as the reference structure.

Among the variables considered for the model of excess zeros, “other insurance” and age were significant. Thus, the chance of presenting excess zero was 9.4 times (95%CI 1.57–55.92) higher in patients with other health insurance (other than the public or private system) in relation to those without insurance. In relation to age, as it increased in one year, the chance to present excess zero decreased by 5.0% (OR = 0.95, 95%CI 0.92–0.98) (Table 4).

**Table 4:** Analysis of the zero-inflated negative binomial model for the relationship between cancer rate and migratory status, adjusted for sociodemographic variables, including additive and interaction effects. Chile, 2012.

Variable		Coefficient	PR	95%CI	p
Negative binomial model (counting)					

Migratory status	Immigrant	0.969	2,6	1.77–3.93	0.000
	Chilean		1		
Age	Age	0.033	1.0	1.02–1.05	0.000
	Age	0.0001	1.0	0.99–1.00	0.343
Sex	Woman	0.095	1.1	0.92–1.32	0.308
	Man		1		
Health insurance	FONASA	0.388	1.5	1.08–2.01	0.014
	ISAPRE	1.071	2.9	2.15–3.96	0.000
	Other	1.568	4.8	3.51–6.56	0.000
	No insurance				
Intersection	Immigrant-FONASA	-2.547	0.8	0.05–0.14	0.000
	Immigrant-ISAPRE	-1.073	0.3	0.20–0.59	0.000
	Immigrant-other	-1.055	0.3	0.17–0.72	0.005
Constant		-7.633	0.001	0.0003–0.0007	0.000

Logistic model (excess zeros)					

Migratory status	Immigrant	20.92	1.22x10^9^	0	0.995
	Chilean		1		
Age	Age	-0.05	0.9	0.91–0.98	0.004
Sex	Woman	0.2	1.2	0.39–3.80	0.73
	Man		1		
Health insurance	Public	-0.13	0.9	0.14–5.47	0.889
	Private	0.4	1.5	0.31–7.24	0.619
	Other	2.24	9.4	1.58–55.92	0.014
	No insurance		1		
Constant		-20.82	0.0	0	0.995

PRR: Prevalence Rate Ratio

In(α) = -0.882 (IC95% -1.120 – -0.644), p = 0.000

Log pseudolikelihood: p = 0.000

Test Vuong: z = 1.70; p = 0.0442

The adjusted ZINB model reaffirms some of the observed results at the level of specific rates. Immigrants without health insurance were 2.6 times (95%CI 1.77–3.93) more likely to leave the hospital because of cancer compared to Chileans without insurance. Other types of insurance had a different situation. Sex was the only variable that had no significant effect on the count model. The adjusted models showed adequate adjustment, supporting the validity of these results (Table 4).

## DISCUSSION

Neoplasms were the third cause of hospital discharges for immigrants (n = 700) and the seventh for the local population (n = 115,406) in Chile in 2012. In the group of immigrants, the CHD mainly corresponded to patients belonging to the private system (46.0%), and in the group of Chileans they mainly corresponded to patients in the public system (71.1%). We observed a large difference in the proportion of CHD for patients with no health insurance between the two populations (22.6%: immigrants, 1.0%: Chileans). In both populations, the three most frequent types of cancer were: (i) lymphoid tissue, hematopoietic organs, and related tissues, (ii) digestive organs, and (iii) breast cancer.

Similar to the results found in Italy for hospital discharges in general^19^, the rate of CHD differs between the local (446.0 CHD per 100,000 persons) and immigrant population (249.3 CHD per 100,000 persons), being it lower for immigrants. Similar to that found in Spain on the total number of hospital discharges^7^, the local population in Chile has longer hospitalization stay for cancer than immigrants. Paradoxically, with some exceptions, for the different types of cancer, sex, and health insurance, immigrants have a higher proportion of surgical interventions for cancer (Figure). All of the above suggests inequalities in the quality, use, and access to secondary health care between both populations.

Similarly to what is happening around the world, changes in breast cancer rates have been observed among working women who migrated to industrialized regions^24^, which highlights the importance of analyzing this pathology in immigrants. In Chile, based on hospital discharges, breast cancer also appears as a focus of special attention in this population. For 2012, the percentage of discharges for cancer within the total HD was higher in immigrants than in Chileans in the same situation. This is one of the only four cancers in which immigrants have a lower proportion of surgical interventions. This reality is similar to that observed in Italy, where immigrants had lower rates of mastectomies than the local population^19^.

This study has strengths, such as: (i) it is the first of its kind in the country^25^, (ii) it can count on the universe of CHD for the local and immigrant population in the period, (iii) it analyzes the exposed problem considering demographic, hospital, and health insurance variables, and (iv) it uses an appropriate analysis methodology^23^, by recognizing the distribution of the studied phenomenon (CHD, their dispersion, and the large number of subgroups in which no CHD is observed) and the difference in the population structures between Chileans and immigrants^3^ (according to phenomena such as the “feminization of migration” and “effect of the health migrant”). This is not only applied at the theoretical level, but it is also proved in practice by the Vuong test, which was significant at a level of 0.05 for the proposed ZINB model. This indicates that this model is a significant improvement to explain the situation of a standard negative binomial model^23^.

This research has limitations, such as: (i) the impossibility of determining in which stage of the disease the HD occurred and if the disease was present before the migratory process, (ii) the difficulty in elucidating whether the results obtained are the product of prevalence of cancer in both populations or whether they were a reflection of the way in which both populations use the health services^26^, (iii) the selection bias, because of the difference in the definition of immigrants in the record of HD (nationality of the patient) and CASEN (“lifetime migration”, mother’s residence at the moment of the person’s birth), and (iv) the information bias when collecting the variable of migratory status in both databases, since it is carried out using different questions (“what is your nationality?”, for hospital discharges, *versus* “in which country did your mother live when you were born?”, for the CASEN database).

This study needs some relevant variables for research. The length of stay in the recipient country is one of the variables that recurrently appears in the literature as fundamental to analyze the different health phenomena in the international migrant population^6^, especially in studies on cancer and more specifically on breast cancer^24^ ^,^ ^27^ ^,^ ^28^. The study by John et al. carried out in the United States in 2005, has indicated that the incidence rate of breast cancer is directly proportional to the time lived in the country, especially in women who had arrived before the age of 20 years^28^. Given the absence of this variable in official records, we could not consider it in the analysis in this research. Similarly, we did not have the variables of socioeconomic status, degree of cultural assimilation, age of arrival in the country, and other variables related to the disease. These variables could be considered in the future for a better understanding of this phenomenon and its implications for public health.

We can identify some implications of this research and public health study. Hospital discharges are a valuable source of information and are usually used to determine morbidity in the population^29^. Similarly, studies on the immigrant population are of great utility for public health and epidemiology, as they compare populations with genetic and ethnic differences that begin to share the social, cultural, and environmental conditions at some point in their life^27^. Along with these premises, the results of this study emphasize the importance of studying cancer in this community, in general, and cancers of interest such as breast cancer. At the same time, studies need to stratify by ethnicity and/or origin, examining the modifiable behavior and environmental determinants before and after the migration process.

This research opens the door for future research in the country for the generation of hypothesis regarding the access and use of tests for the early detection and timely treatment of cancer by immigrants. Cancer is a disease in which survival tends to be worse when factors such as social vulnerability, late diagnosis, and access limitations are combined, which fits the profile of some immigrant groups in Chile and Latin America.

## Acknowledgments

The authors would like to thank the department of health statistics and information (DEIS) of the Ministry of Health of Chile for delivering the database of hospital discharges of 2012.
